# The Effect of Health Belief Model Educational Program and Jogging on Control of Sugar in Type 2 Diabetic Patients

**Published:** 2012-07-30

**Authors:** S M Kashfi, A Khani Jeihooni, A Rezaianzadeh, Sh Amini

**Affiliations:** 1 Department of Public Health, School of Health and Nutrition, Health Science Research Center, Shiraz, Iran; 2 Department of Public Health, Fasa University of Medical Sciences, Fasa, Iran; 3 Department of Epidemiology, Shiraz University of Medical Sciences, Shiraz, Iran; 4 Shiraz University of Medical Science, Shiraz, Iran

**Keywords:** Type 2 DM, Jogging, Health Belief model

## Abstract

**Background:**

Researchers believe that most of diabetic patients are not necessarily aware of the role of exercises, especially jogging in controlling their disease. The purpose of this study was to evaluate the effect of educational program and jogging based on health belief model (HBM) on sugar control in type 2 diabetic patients.

**Methods:**

One hundred diabetic (type 2) patients were involved in this prospective quasi-experimental interventional study. Patients were randomly divided into two groups of experimental and control. Data was collected using a questionnaire based on the HBM model, a check list for patient`s practices and a check list for recording the patient`s hemoglobin (HbA1C) and fasting blood sugar (FBS) levels.

**Results:**

Our findings indicated that after intervention, there was a significant difference between the mean score of the HBM model variables (susceptibility, severity, benefit and perceived obstacles,) in the experimental group compared to the control group. Additionally, behavioral jogging, level of HbA1C and FBS levels improved significantly among the experimental group when compared to the control group.

**Conclusion:**

Applying the HBM model was found to be a very effective means for developing an educational program of jogging for diabetics, in order to control their blood sugar.

## Introduction

Today, specialists believe that diet and medication are not enough to treat and control sugar in diabetic patients alone, but exercises and activities should also be added to the daily schedule of diabetic patients.[1] Useful exercises for diabetic patients include jogging, cycling, swimming, etc.[2] Jogging is a simple and worthwhile exercise and it is possible to include it in daily activities of diabetic patients, regardless of their age and gender, without a need for a specific equipment.[1][3] Jogging has remarkable health effects on all age groups including sensitizing cells to insulin, weight loss, decreasing lipid tissue, fall in blood pressure, increasing physical fitness and causing mental exhilaration and in less serious cases of disease, probability a decrease or elimination of drugs for type II diabetic patients.[3][4] Also inactivity doubles the risk of cardiovascular diseases in diabetic patients.[5] Various studies indicated that a large percentage of type II diabetic patients in our society were inactive or did very light activities in their leisure time.[6]

Researchers believe that most of diabetic patients are not necessarily aware of the role of exercises, especially jogging in controlling their disease desirably and do not use this important therapeutic principle enough.[2] Thus, training the patients to jog continuously based on patterns which identify and reinforce the effective factors seems necessary. This study was performed to evaluate the effect of educational program and jogging, based on health belief model (HBM) on sugar control in type 2 diabetic patients.

## Materials and Methods

One hundred diabetic (type 2) patients were involved in this prospective quasi-experimental study. Diabetic patients were those without diabetic foot and cardiovascular side effects and were randomly selected from a diabetes center, Ahmad Nader Kazemi Clinic and they were randomly divided into two groups of experimental and control. Data was collected using a standard questionnaire including data on demographic characteristics, knowledge, dimensions of health belief model including perceived obstacles, interests, intensity and sensitivity, and also data regarding jogging. In addition, the amount of fasting blood sugar (FBS) and glycoside hemoglobin (HbA1c) of the patients before and three months after the training intervention were recorded. The intervention was 3 sessions (each 60 minutes) of training on jogging and control of sugar. Reliability of the questionnaire was reviewed by means of taking a reexamination and it was certainly defined 84% and its faults were also eliminated. For understanding 5-choice questions and questions related to the dimensions of health belief model, Likert 5-choice theory meter scale was used and the time period of jogging of patients was also measured during the last week based on minute.

Ethical approval for this study was obtained from Research Ethical Committee of Shiraz University of Medical Sciences. Data analysis was performed using SPSS software (Version 15, Chicago, IL, USA). Chi square test, independent and pair-T test were used. Significance level was considered as 0.05.

## Results

Mean age and its standard deviation in experimental and control groups was 43/9±4/9 and 45/1±3/8 years respectively. Mean duration of affecting by type II diabetes in experimental group was 5/45±3/28 years. Subjects were mainly married and had elementary educational level (Table 1).

**Table 1:** Relative frequency distribution of persons according to the study, marital status, occupation and education

**Demographic variables**	****	**Case group**		**Control group**		**Total**	
****	****	**No.**	**(%)**	**No. **	**(%)**	**No.**	**(%)**
Marital status	married	47	94	45	90	92	92
	single	3	6	5	10	8	8
Chi-square Test	*P*=0.308						
Education	Illiterate	10	20	10	20	20	20
	Elementary	16	32	16	32	32	32
	guidance	10	20	11	22	21	21
	Diploma	14	28	13	26	27	27
Chi-square Test	*P*=0.904						

Before intervention, no significant difference was detected between two groups. However, after interventions all variables were significantly different (Table 2). Three months after the end of training program, experimental group showed better instructions for jogging in comparison to the control group. While the amount of HbA1c (from 8.95 to 7.90 Unit? in three months after the training intervention) and blood sugar (from 180 before the intervention to147.5 mg/dl in three months after the training intervention) had a remarkable decrease and it was statistically significant (p<0.001). In addition, before training intervention, most of internal instructions of patients for jogging in the group of intervention and control respectively included feeling exhilaration during jogging (50% and 47%), fear of blood sugar increase (38% and 34%) and fear of catching side effects of diabetes (36% and 40%). But after training intervention, Chi Square test indicated a significant difference in all the internal instructions of diabetic patients for jogging (Table 3).

**Table 2:** Comparison of knowledge and sensitivity and severity and threat and perceived benefits and barriers mean and performance of right jogging, fasting blood sugar and trimester HbA1c glycosulated hemoglobin mean of patients before and 3 month after the educational intervention between case and control groups.

**Desired variable**	**Group**	**Before intervention**		**After intervention**	
		**Mean**	**Standard deviation**	**Mean**	**Standard deviation**
Awareness	case	53.1	21.6	80	6.8
	control	53.1	21	55.9	9.4
	Independent t-test	*P*=0.740		*P*<0.001	
Perceived sensitivity	case	52.87	17.91	73.71	7.8
	control	51.31	15.53	51.08	9.11
	Independent t-test	*P*=0.634		*P*<0.001	
Perceived severity	case	50.98	16.89	74.16	3.17
	control	53.1	17.31	56.36	10.13
	Independent t-test	*P*=0.562		*P*<0.001	
Perceived threat	case	51.93	13.65	73.93	4.19
	control	51.97	14.09	53.79	9.5
	Independent t-test	*P*=0.988		*P*<0.001	
Perceived benefits	case	62.72	14.61	94.46	4.17
	control	66.81	12.02	67.95	7.99
	Independent t-test	*P*=0.155		*P*<0.001	
Perceived barriers	case	55.52	15.82	33.40	3.84
	control	57.30	11.96	55.15	9.5
	Independent t-test	*P*=0.566		*P*<0.001	
Performance of right jogging	case	37.31	30.73	75.94	8.35
	control	36.17	29.1	39.72	6.73
	Independent t-test	P=0.859		P<0.001	
Fast blood sugar (mg/dl)	case	180	44.66	147.5	29
	control	175.25	46.08	170.12	36.5
	Independent t-test	P=0.520		P<0.001	
trimester glycosulated hemoglobin (HbA1c)	case	8.95%	0.64	7.90%	0.54%
	control	8.91%	0.89	8.77%	0.68%
	Independent t-test	P=0.411		P<0.001	

**Table 3:** Frequency distribution of jogging performance based on patients comments after the educational intervention in both case and control groups.

**Study groups**	**Case**		**Control**		**Chi-square test result**
	**No.**	**%**	**No.**	**%**	
Increased blood sugar	28	63%	17	38%	P<0.001
Status of general situation	22	50%	13	13%	P<0.001
Fear of diabetes complications	30	68%	17	17%	P<0.001
Happy feel if doing jogging	36	81%	25	25%	P<0.001

## Discussion

The mean score of knowledge was similar between two groups before intervention. However, after intervention, two groups were significantly different in this regard. This results were consistent with results of two other studies.[7][8] The existence of significant difference between the average of knowledge grades of experimental and control groups after the training intervention can be known as the reason of holding training classes on jogging which the knowledge in experimental group to the grade of 25.37 for proper jogging. These findings accords with other research.[9][10] In addition, findings indicate that the average status of perceived sensitivity grade of patients was significantly different between two groups. Two other studies showed similar results.[11][12] Before the training intervention, the results indicate that patients' perception status of the benefits of jogging in both groups was a little more than average level which increased more in experimental group after the training intervention (31.74 grade). This increase in grade in the experimental group was 30 times more than the control group. Study by Kouch (2002) also shows that in type II diabetic women, there is a positive relation between perceived benefits and jogging.[13] Study by Robinson-Whelen and Bodenheimer (2004)[14] and a study in Thailand [15] also indicated a significant relationship between perceived benefits and doing exercises.[14][15] In addition, findings by Kamrani (2006) and Asghari (2006) in the section of perceived benefits resembles with the present research.[16][17]

In this study, a significant difference in perceived obstacles between the two groups after the training intervention indicated the effect of education on removing perceived obstacles. This result is identical to other studies.[18][19] In the present study, the average of time period of jogging in the experimental group has been 97 minutes and in control group 94 minutes during a week which showed no significant difference.

In the study by Forghani et al. (2000), the average jogging for type II diabetic patients of Isfahan Diabetes Center per week was reported 124 minutes which is more in comparison with the current study. In the present research as it is mentioned in Table 2, the main part of the amount of jogging (78%) did not have positive results for the patients. After the training intervention in the experimental group, the average of days of jogging has been increased to 4.70 days in a week and the average of jogging minutes has also increased to a remarkable amount and reached 226 minutes in a week. Our findings showed that it is required to go jogging at least three days a week and each time for 30-40 minutes, so the obtained results is the indicator of the positive effect of educational intervention in this area. As it is observed in Table 2, the percentage of jogging with low intensity had no effects on disease recovery, while decreased from 76% before educational intervention to 36% after the intervention, but the amount of jogging cases with average and high intensity increased which is an indicator of the positive effect of educational intervention.

Based on the information in Table 2, the average fasting blood sugar (FBS) in patients of experimental and control group before educational intervention, has been 180 mg/dl and 175.25 mg/dl respectively which showed no significant difference with each other. This finding is similar to the results of others.[19][20]

In some studies after the educational intervention, the average amount of blood sugar before breakfast decreased in patients.[16][21] The average of HbA1c before the educational intervention for the experimental group was 8.95% and for the control group was 8.91% which show no significant difference. Another study also showed similar results.[10] After the educational intervention, the average HbA1c of the patients in the experimental group reached to 8.63% and in the control group 9.37%. The result of this study similar to other studies showed that exercise and weight loss were effective and useful for decreasing blood sugar and helped to control their blood sugar for long-term in diabetic fat people.
